# All Biomass‐Based Solar‐Driven Interfacial Evaporator for Efficient Seawater Desalination and Power Generation

**DOI:** 10.1002/advs.202513258

**Published:** 2025-09-25

**Authors:** Jinlong Zhu, Changyou Shao, Sanwei Hao, Jifei Zhang, Wenfeng Ren, Bing Wang, Ling‐Ping Xiao, Hanhui Lei, Terence X. Liu, Zhanhui Yuan, Runcang Sun

**Affiliations:** ^1^ Liaoning Key Laboratory of Lignocellulose Chemistry and BioMaterials Liaoning Collaborative Innovation Center for Lignocellulosic Biorefinery College of Light Industry and Chemical Engineering Dalian Polytechnic University Dalian 116034 China; ^2^ School of Materials Science and Engineering Shandong University of Technology Zibo 255000 China; ^3^ Department of Mechanical and Construction Engineering Northumbria University Newcastle upon Tyne NE1 8ST UK; ^4^ College of Materials Engineering Fujian Agriculture and Forestry University Fuzhou 350002 China

**Keywords:** aerogel, biomass, cellulose, hydrogel, lignin, solar‐driven interfacial evaporator

## Abstract

Solar‐driven interfacial evaporation has emerged as a promising strategy to alleviate global water scarcity. However, most existing evaporators still depend on synthetic materials, raising concerns over their environmental impact. Herein, Fe^3^⁺‐coordination‐driven assembly of cellulose nanofiber (CNF)/Fe^3^⁺/lignosulfonate (LS) into photothermal layer (CFL) with a porous CNF/Fe^3^⁺ aerogel (CF) water transport channe, respectively, to construct a fully biomass‐derived solar‐driven interfacial evaporator. The results establish that Fe^3^⁺‐LS coordination synergistically endows with the outstanding photothermal performance of the CFL hydrogel, achieving 95% full‐spectrum solar absorption. The hydrated polymer network present in the CFL hydrogel can regulate the molecular state of water, effectively lowering its vaporization enthalpy while maintaining rapid water transport. Based on these advantages, the system achieves an evaporation rate of 1.91 kg m^−^^2^ h^−1^ under 1 sun. The fully biomass‐based evaporator can remove ≈96.6% of major metal ions (Na⁺, K⁺, Ca^2^⁺, Mg^2^⁺) from seawater to generate fresh water, exhibiting superior salt resistance and durability. Notably, CFL hydrogels integrated with a thermoelectric module achieve an open circuit voltage of 110 mV, enabling direct operation of a commercial fan. This work develops an environmentally benign and cost‐efficient strategy employing a fully biomass‐derived hydrogel evaporator from renewable plant‐based bioploymers for sustainable seawater desalination.

## Graphical abstract

A fully biomass‐derived hydrogel for solar interfacial evaporation is engineered through Fe^3+^‐coordinated assembly of cellulose nanofibril (CNF) and lignosulfonate (LS). This sustainable evaporator achieves a high evaporation rate of 1.91 kg m^−2^ ^h−1^ under 1 sun irradiation and demonstrates efficient desalination. The system showcases exceptional salt resistance and durability, offering a promising path for sustainable fresh water production.

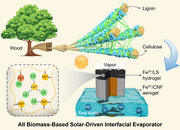

## 1 Introduction

Freshwater scarcity is one of the sternest global issues today.^[^ ^1^ ^]^ While water covers ≈75% of Earth's surface, only 2.5% exists as freshwater, with nearly 2 billion people currently suffer from freshwater shortages. Various desalination approaches, such as reverse osmosis, electrodialysis, freezing, and multi‐stage flash, offer diverse pathways to freshwater production.^[^ ^2^ ^]^ However, many of these methods rely on external devices and are driven by secondary energy sources such as thermal or electric energy, which frequently depend on fossil fuels or other nonrenewable resources. In contrast, solar energy emerges as the most abundant and sustainable renewable resource, offering a clean alternative that circumvents both carbon emissions and the inefficiencies of energy conversion.^[^ ^3^ ^]^ Consequently, solar‐driven desalination technology is considered a green technology uniquely positioned to address the global water crisis while aligning with sustainable development goals.^[^ ^4^ ^]^ This potential has driven rapid advances in solar‐driven desalination, particularly interfacial evaporation systems that concentrate thermal energy at the air‐liquid interface. By localizing heat to the evaporation surface, these systems achieve superior energy efficiency compared to conventional bulk heating methods, positioning them as a promising solution for sustainable water production.^[^ ^5^ ^]^ The inherent temperature gradient between the evaporative surface and bulk water can be exploited for thermoelectric power generation via the Seebeck effect, enabling dual‐output functionality within a single integrated device.^[^ ^6^ ^]^ By unifying solar steam generation, water purification, and electricity production, such systems present a synergistic solution to water stress and energy scarcity. This multifunctional approach has thus attracted growing interest as a sustainable strategy for resource co‐generation.

Hydrogel‐based interfacial solar evaporators have emerged as a promising platform for sustainable water purification. These systems typically combine a hydrophilic polymer matrix with photothermal materials, where the hydrogel network both reduces evaporation enthalpy and maintains continuous water supply to the evaporation interface.^[^ ^7^ ^]^ Current designs of polymer matrix predominantly employ synthetic polymers like polyvinyl alcohol,^[^ ^8^ ^]^ raising concerns about environmental persistence and end‐of‐life management. This limitation has driven interest in nanocellulose as a sustainable alternative, which is the most abundant biopolymer in nature and derived from plants, fungi, algae, and bacteria.^[^ ^9^ ^]^ Nanocellulose offers unique advantages for evaporator design: (1) surface hydroxyl groups enabling versatile chemical modification; (2) hydrogen‐bonded networks providing mechanical reinforcement through dual crosslinking effects; and (3) the ability to form highly porous aerogels and hydrogels with exceptional surface‐area‐to‐volume ratios.^[^ ^10^ ^]^ These properties make cellulose‐based material platforms particularly interesting for use as structural elements in solar‐driven interfacial evaporator.

Beyond substrate optimization, significant research efforts have focused on photothermal materials for interfacial solar evaporation. A variety of photothermal materials have been reported, ranging from noble metal nanomaterials,^[^ ^11^ ^]^ transition metal materials,^[^ ^12^ ^]^ carbon‐based nanomaterials,^[^ ^13^ ^]^ organic conjugated materials,^[^ ^14^ ^]^ While these materials have demonstrated excellent solar energy absorption and photothermal conversion properties, their practical application is substantially limited by complex synthesis, high costs, and environmental concerns. This has spurred interest in developing evaporators that combine high evaporation rates with long‐term salt resistance using exclusively low‐cost, renewable biomass. Lignin, a major plant cell wall component comprising cross‐linked phenylpropane units, presents an attractive solution.^[^ ^15^ ^]^ Its rich aromatic ring structures, aliphatic and aromatic hydroxyl groups, and quinone groups, enable strong π–π stacking,^[^ ^16^ ^]^ promoting nonradiative migration and triggers photothermal conversion.^[^ ^17^ ^]^ Moreover, the low thermal conductivity of lignin provides excellent thermal insulation, minimizing heat loss to the bulk water.^[^ ^18^ ^]^ Furthermore, when sulfonated to form lignosulfonate (LS), these conjugated structures are further functionalized with sulfonate groups, which enhance hydrophilicity while retaining the original π‐conjugated backbone.^[^ ^19^ ^]^ The hydroxyl groups in LS establish noncovalent interactions with surrounding water molecules, altering the hydrogen‐bonding network and thereby regulating the state of water, which facilitating the formation of intermediate water.^[^ ^20^ ^]^ This mechanism plays a pivotal role in enhancing the evaporation efficiency of biomass‐derived hydrogel evaporators. Despite these advantages, only ≈2% of the 50 million tons of LS produced annually from pulping processes are used for high‐value products, with the remainder being burned as low‐value fuel.^[^ ^21^ ^]^ Its deployment in solar evaporators could therefore simultaneously address water scarcity challenges while creating a sustainable pathway for this underutilized biomass.

Herein, we developed an Fe^3+^‐coordinated, entirely biomass‐derived evaporator using CNF as the structural scaffold and LS as the photothermal component, with broadband absorption of sunlight and an exceptional water evaporation rate. The 3D structure of the evaporator was fabricated through the separation of water supply channels and the evaporative layer, coupled with foam‐based insulation to minimize heat dissipation. A hydrophilic and porous aerogel ensures rapid water transport, enabling sustained evaporation under solar irradiation. The bandgap of hydrogel reduction via LS‐Fe^3^⁺ coordination broadens its solar absorption spectrum. Simultaneously, the hydrophilic network of hydrogel reduces evaporation enthalpy and thereby enhances evaporation rate. As a result, the fully biomass‐based evaporator exhibited an excellent water evaporation rate of 1.91 kg m^−2^ h^−1^, surpassing most previous biomass‐based evaporators. Remarkably, when coupled with a thermoelectric module, CFL hydrogels exhibit an open‐circuit voltage output of 110 mV‐sufficient to drive a commercially available fan without additional power conditioning. Therefore, this study demonstrates the possibility of realizing high value practical applications biomass resources in the fields of high‐efficiency solar interfacial evaporation, seawater desalination, and power generation.

## 2 Results and Discussion

**Figure 1:**
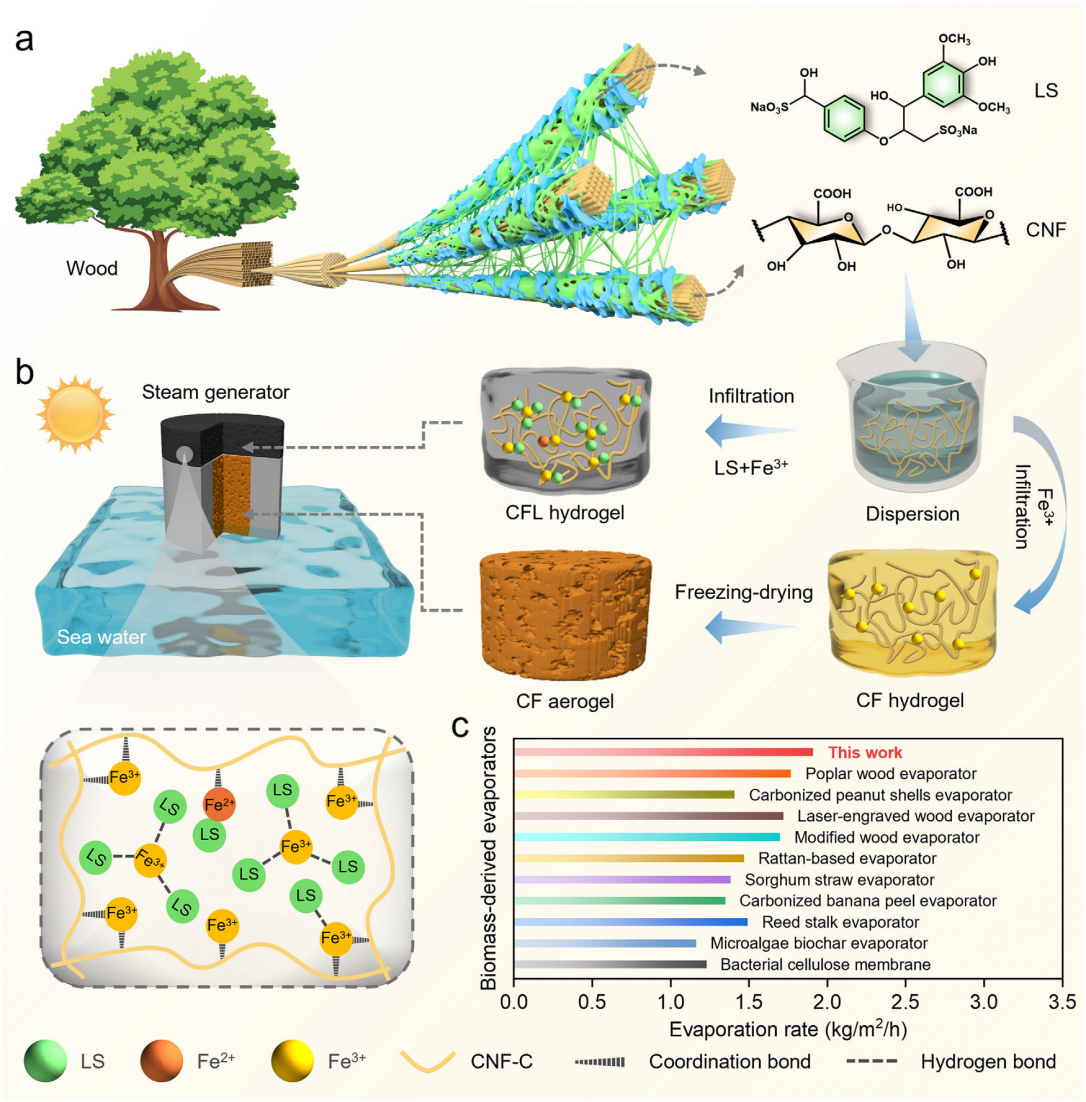
a) Schematic of the source of materials. b) a process flow chart of device fabrication. c) comparison of the water evaporation rates with previously reported biomass‐derived evaporators under one sun irradiation.

CNF consist of *β*1,4‐linked D‐glucose monomers, where each glucose unit bears two highly reactive hydroxyl groups and a carboxyl group (**Figure** **1**a). Here, we demonstrate that Fe^3^⁺ coordination with carboxyl groups facilitates CNF gelation (CF hydrogel), which, upon freeze‐drying, yields a lightweight aerogel (CF aerogel). Owing to its abundance of hydrophilic functional groups (‐OH and ‐COOH) and an intrinsically porous architecture, the resulting CNF aerogel exhibits exceptional hydrophilicity. Moreover, the freeze‐drying process introduces microscale surface roughness, further enhancing wettability.^[^ ^22^ ^]^ To enable photothermal functionality, we incorporate LS, an amorphous polymer consisting of cross‐linked benzene rings connected through heterogeneous C─O─C and C─C bonds.^[^ ^23^ ^]^ The aromatic structure features abundant phenolic hydroxyl groups, which provide abundant binding sites for chemical modification.^[^ ^24^ ^]^ By immersing the CF hydrogel in a mixed solution of LS and Fe^3+^, the CFL hydrogel was obtained (Figure 1a). Notably, coordination between Fe^3^⁺ and these hydroxyl groups of LS induces a visible darkening of the CFL hydrogel, significantly enhancing its broadband solar absorption properties. In addition, The LS chain contains a significant quantity of sulfonate and hydroxyl groups,^[^ ^25^ ^]^ these hydrophilic moieties establish extensive hydrogen‐bonding networks with water molecules, effectively restructuring the aqueous phase to favor intermediate water formation^[^ ^26^ ^]^—a critical factor in enhancing evaporation rates in solar‐driven hydrogel evaporators. Additionally, LS featured with reducing the methoxy groups and phenolic hydroxyl groups enables to be converted into the quinone/catechol/semiquinone with oxidation or reduction activities.^[^ ^27^ ^]^

Leveraging the excellent hydrophilicity and light absorption ability of these gels, we employed the CF aerogel as the water‐transport pathway and the CFL hydrogel as the photothermal absorption layer. And a rough surface texture on the solar absorber was obtained through template‐assisted modulation using abrasive paper. As illustrated in Figure 1b, the two structural elements and the foam float, were integrated and constructed as a 3D solar‐driven interfacial evaporator (see details in the “Experimental Section”). The buoyancy generated by the float supports the entire solar evaporator, effectively isolating the solar absorption layer above the water surface. Hydrophilic CNF networks enable efficient water transport through capillary driven, while the LS‐Fe^3^⁺ composite photothermal layer achieves high‐efficiency solar vapor generation. A systematic investigation of the preparation ratios was conducted to optimize the evaporation rate, as detailed in the Figures S1–S3 (Supporting Information). This integrated system demonstrates continuous water supply and vapor production under sunlight. In this contribution, which distinguishes this study from many previous reports. The all‐natural material composition‐combining the structural integrity of CNF with the photothermal properties of LS‐establishes a sustainable platform for interfacial solar vapor generation. Systematic evaluation demonstrates that this all‐biomass‐based evaporator achieves competitive solar steam generation performance among reported biomass‐derived systems, while exhibiting exceptional operational durability and environmental compatibility, as shown in Figure 1c and Table S1 (Supporting Information).

**Figure 2:**
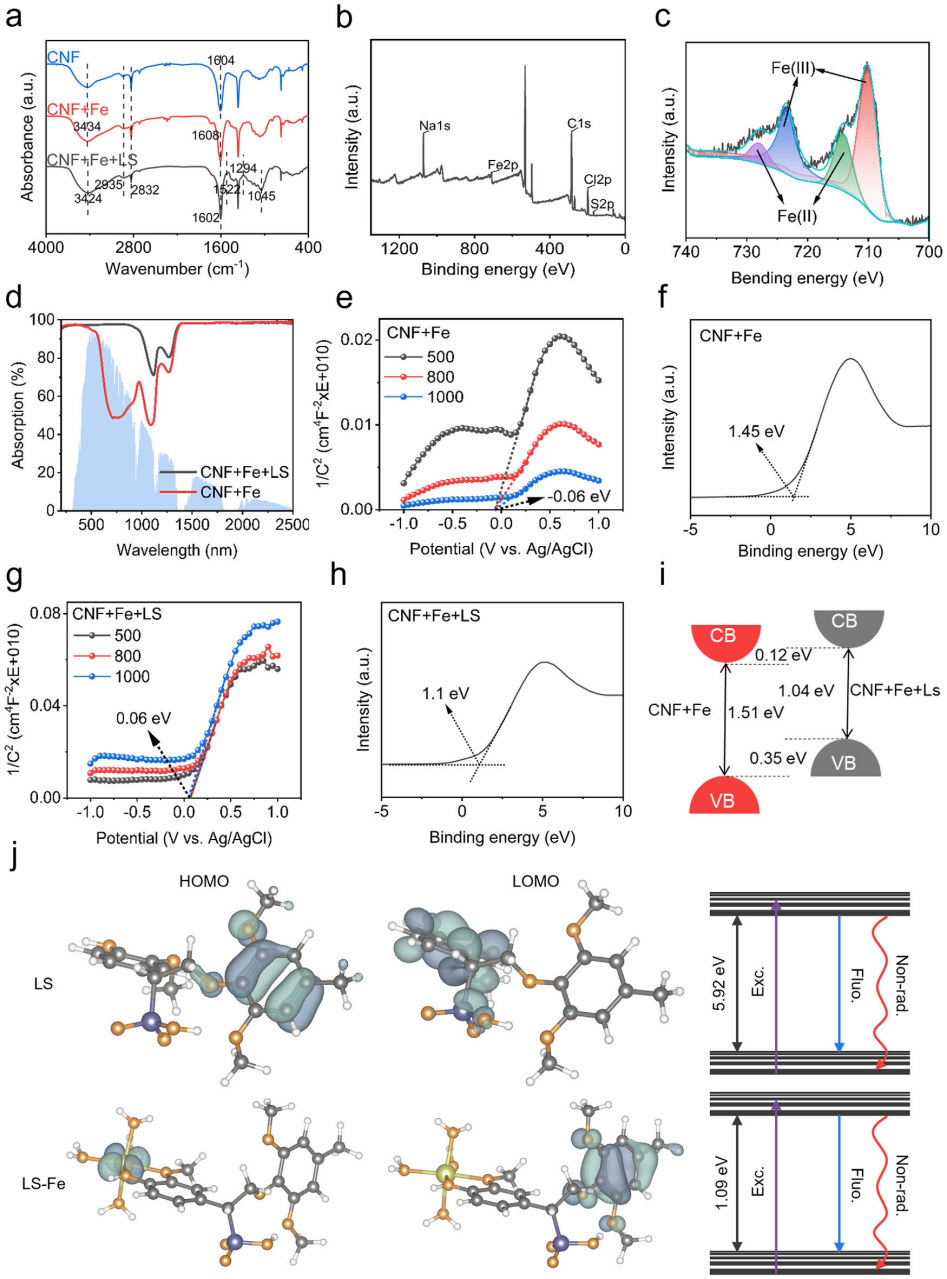
a) FTIR spectra of CFL hydrogel and CF aerogel. b) XPS of CFL hydrogel. c) XPS of Fe, d) UV–vis–NIR absorption spectrum for CFL hydrogel and CF hydrogel. e,g) Mott‐Schottky curves of CF aerogel and CFL hydrogel. f,h) valence band (VB)‐XPS of CF aerogel and CFL hydrogel. i) energy band structure scheme of CF aerogel and CFL hydrogel. j) calculated energy and molecular orbits of LS and LS‐Fe^3+^.

FTIR spectroscopic analysis verifies the successful formation of CF aerogel and CFL hydrogel (**Figure** **2**a). In the range of 3000–3700 cm^−1^, all of the spectra show strong wide absorption, indicating that a significant number of hydroxyl groups is present in these samples. The peak at 3434 cm^−1^ corresponds to O‐H stretching vibration. An obvious redshift of CFL hydrogel from 3434 to 3424 cm^−1^ was also observed, which demonstrated the complexation of coordination bonds in composites.^[^ ^28^ ^]^ The double peaks at 2935 and 2832 cm^−1^ in all of the spectra correspond to the C─H stretching vibration. In CNF sample, the asymmetric stretching vibration of carboxylate groups generates a characteristic peak at 1604 cm^−1^. Upon incorporation of Fe^3^⁺, this peak shifts distinctly to 1608 cm^−1^ in the CF sample and to 1602 cm^−1^ in the CFL sample, confirming Fe^3^⁺–carboxylate coordination. CFL hydrogel showed a peak at 1522 cm^−1^, belonging to the stretching vibrations of benzenoid rings. For the CFL hydrogel, the presence of LS results in a notably strong peak at 1045 cm^−1^, which is attributed to the S═O stretching vibrations.^[^ ^29^ ^]^ The XPS spectra of CFL hydrogel was performed in Figure 2b to show the detailed structural information. The Fe 2p spectrum of CFL hydrogel showed the Fe 2p peak at 710.05 eV, demonstrated that Fe^3+^ had successfully existed on the CF aerogel. The peak of Fe 2p had shifted, further illustrating the presence of coordination effect between LS and Fe^3+^. The peak of Fe^2+^ was discovered because of the redox property of LS (Figure 2c; Figure S4, Supporting Information).

The absorption of sunlight is the initial step in the interface evaporator for photothermal evaporation, making the selection of photothermal materials particularly critical. An ideal photothermal material should achieve a high light absorption rate across the entire solar spectral range. The solar absorption characteristics were investigated using ultraviolet‐visible‐near infrared (UV‐vis‐NIR) spectroscopy over a wide range of wavelengths from 200 to 2500 nm. As shown in Figure 2d, CFL hydrogel exhibits a broad absorptance of ≈95% in the wavelength range of 250–2500 nm, which is higher than that of CF aerogel (≈86%), demonstrating that LS can improve solar absorption due to its abundant aromatic rings and π‐π conjugated structure.^[^ ^30^ ^]^ Figure 2e,g, the conduction band (CB) of CF aerogel and CFL hydrogel was determined to be ‐0.06 V (vs Ag/AgCl) and 0.06 V (vs Ag/AgCl), respectively. As shown in Figure 2f,h, the VB of CF aerogel and CFL hydrogel were 1.45 and 1.1 eV, respectively. Based on the CB and VB, the relative band structure scheme of CF aerogel and CFL hydrogel is shown in Figure 2i. A much narrower bandgap was obtained for CFL hydrogel (1.04 eV) than for CF aerogel (1.51 eV), which agrees with the results obtained by spectral absorption. Because the less energy is required to excite electrons from the valence band to the conduction band when the bandgap is smaller.^[^ ^31^ ^]^ Through computational analysis of the molecular orbital energetics, we reveal a modulation of the electronic structure upon Fe^3^⁺‐LS coordination (Figure 2j). For LS, the highest occupied molecular orbital (HOMO) and lowest unoccupied molecular orbital (LUMO) energies are −6.51 and −0.59 eV, respectively, corresponding to a wide band gap of 5.92 eV. In contrast, the energy levels of the LUMO and HOMO of Fe^3^⁺‐LS coordination are −4.80 and −5.89 eV, reducing the band gap to 1.09 eV. The narrowed band gap promotes electron excitation, thereby enhancing light absorption capacity. The strong agreement between computational and experimental results further confirms that Fe^3^⁺‐LS coordination serves as an effective strategy for optimizing lignin's photothermal performance. Collectively, when exposed to light, the CFL hydrogel undergoes photon absorption, promoting electrons from the valence band to the conduction band. These excited electrons undergo non‐radiative transitions, releasing energy in the form of heat during this relaxation process. Crucially, the coordination of LS with Fe^3^⁺ lowers the bandgap of CFL hydrogel, enabling absorption of lower‐energy photons and thus expanding its effective spectral range.

**Figure 3:**
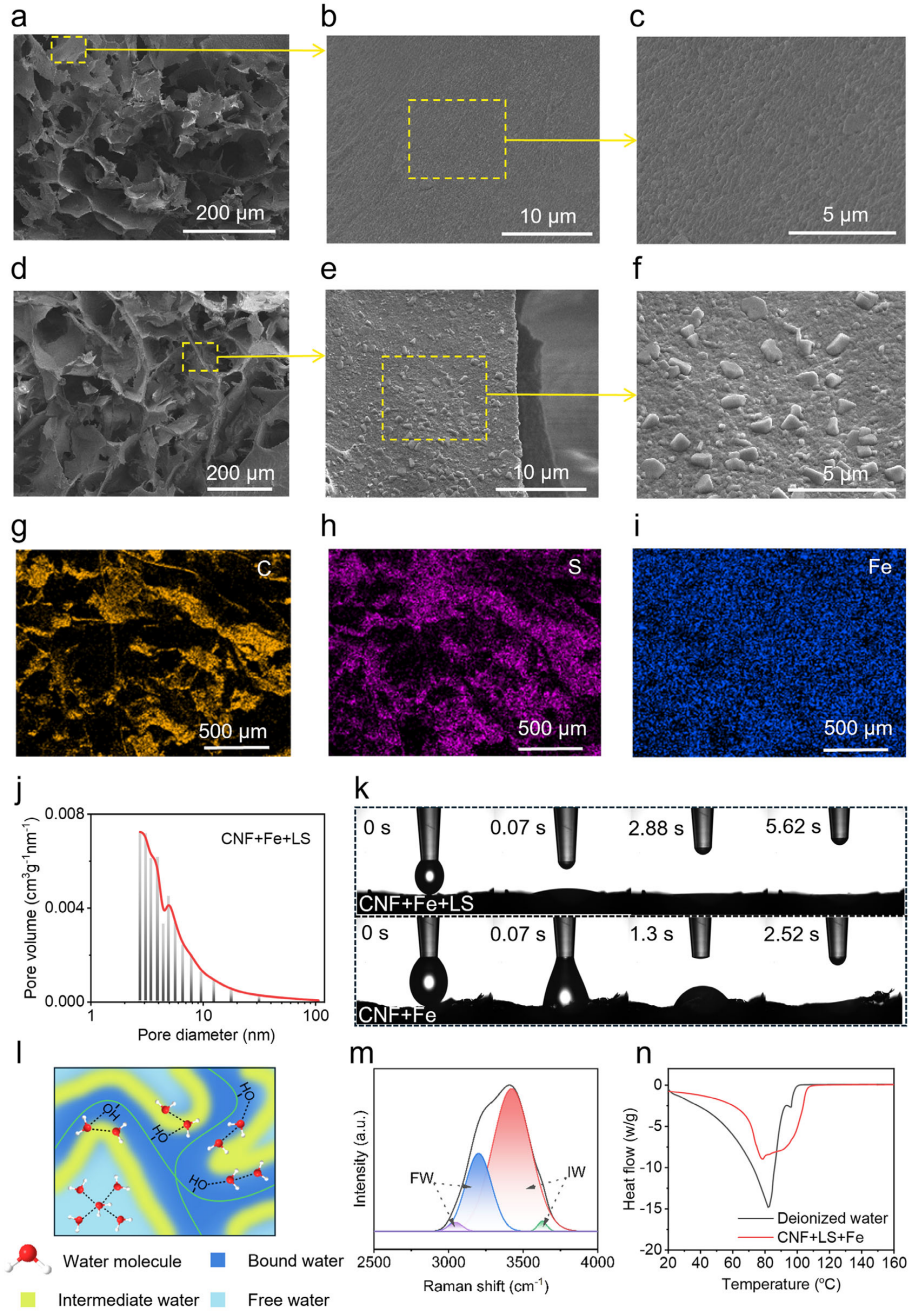
a‐c) SEM images of the interior view of CF aerogel with different magnifications. d‐f) SEM images of the internal structure of CFL hydrogel with different magnifications. g‐i) EDS mapping of the CFL hydrogel. j) the pore size distribution curves of CFL hydrogel. k) optical image of the water dropped on the surface of CFL hydrogel and CF aerogel. l) schematic of the water in the hydratable polymer network of the CFL hydrogel, showing water/polymer bonding (Bound water, BW), weakened water/water bonding (Intermediate water, IW), and normal water/water bonding (Free water, FW). m) raman spectra showing the fitting peaks representing IW and FW in the CFL hydrogel. n) differential scanning calorimetry (DSC) curves of the CFL hydrogel and DI water.

Scanning electron microscopy (SEM) analysis of freeze‐dried samples revealed distinct microstructural features of the gel (**Figure** **3**). Both the CFL hydrogel and CF aerogel exhibited highly porous 3D networks, thereby contributing to the adsorption and diffusion of water molecules (Figure 3a). High‐resolution imaging reveals a well‐ordered distribution of Fe^3^⁺ particles within the CF aerogel matrix (Figure 3b,c), suggesting a structurally uniform aerogel. Microstructural analysis reveals that the CFL hydrogel exhibits a highly porous architecture, with pore diameters spanning 1–100 µm (Figure 3d). Remarkably, this macroporous network remains visible at the macroscopic scale prior to microscopic examination. Moreover, the enhanced structural integrity of the interconnected pores—compared to the CF aerogel—can be ascribed to the incorporation of LS, which serves to reinforce the gel's pore framework and confer improved mechanical robustness. The presence of LS and Fe^3^⁺ is clearly discernible at the edges of the pores (Figure 3e). Fe^3^⁺ and LS showing stochastic dispersion patterns with LS preferentially forming micron‐scale aggregates of irregular morphology (Figure 3f). The pore walls exhibit pronounced surface roughness, which serves to enhance light‐harvesting efficiency by promoting photon scattering.^[^ ^32^ ^]^

Elemental mapping at the microstructural level, carried out using SEM coupled with energy dispersive X‐ray spectrometry (EDS), demonstrates a uniform distribution of C, S, and Fe throughout the CFL hydrogel (Figure 3g‐i). This observation indicates that Fe^3^⁺ is effectively cross‐linked with the CNF, contributing to the stability of the hydrogel's structure. The presence of LS is also confirmed by EDS analysis. Notably, the EDS images also reveal distinct pores within the hydrogel (Figure 3g,h), consistent with the inherent porous nature of the CNF network. Moreover, elemental mapping of the CF hydrogel reveals a homogeneous distribution of Cl, C, Fe, and O (Figure S5, Supporting Information), further underscoring the material's stable and well‐defined porous architecture.

SEM provided initial insights into the presence of micropores within the hydrogel. To further characterize the pore structure, a surface area analyzer was employed, revealing that the pore sizes of the CFL hydrogel and CF aerogel range from 2 to 110 nm (Figure 3j; Figure S6, Supporting Information). The incorporation of micropores within the gel matrix increases the overall surface area, thereby enhancing the adsorption capacity and modulating the solute diffusion rate. These features are anticipated to improve hydrogel's resistance to salt. Both the CF aerogel and CFL hydrogel exhibit type IV isotherms (Figure S7, Supporting Information). Notably, the specific surface area of the CFL hydrogel (25.45 m^2^ g^−1^) is substantially higher than that of the CF aerogel (15.68 m^2^ g^−1^) (Figure S8, Supporting Information).

Hydrophilicity is a critical factor ensuring continuous water transport by the evaporator to the surface for evaporation. To evaluate the hydrophilicity of the CFL hydrogel and CF aerogel, water contact angle measurements were performed. Upon deposition of water droplets onto the surfaces of the CFL hydrogel and CF aerogel, the droplets rapidly diffused and were completely absorbed within 5.62 and 2.52 s, respectively (Figure 3k). These results highlight the strong hydrophilic character of both materials, which arises from the combination of their highly porous structures and the presence of abundant hydroxyl and carboxyl groups in the CNF. Comparative measurements on the CF hydrogel revealed that water droplets were fully absorbed after 6.19 s (Figure S9, Supporting Information), indicating a slower absorption rate relative to the CFL hydrogel. This difference may be attributed to the enhanced hydrophilicity imparted by the incorporation of LS in the CFL hydrogel. Capillary rise tests further demonstrated that water ascended to a height of 6 cm within 100 s, confirming that the CF aerogel is capable of supporting efficient water transport (Figure S10, Supporting Information).

To elucidate the state of water within the CFL hydrogel, we classified water in the hydrated polymer network according to differences in intermolecular hydrogen bonding, including water/polymer bonding, weakened water/water bonding, and normal water/water bonding (Figure 3l).^[^ ^33^ ^]^ Based on this classification, three distinct types of water were identified: free water (Figure 3l, FW, light blue color), intermediate water (Figure 3l, IW, yellow color), and bound water (Figure 3l, BW, dark blue color), respectively. We analyze the raman spectra in the region of O‐H stretching to show the hydrogen bonding distinction of water molecules in the CFL hydrogel, revealing the water state in the gel (Figure 3m). The peaks at 3044 and 3240 cm^−1^ correspond to FW with four hydrogen bonds (two protons and two lone electron pairs are involved in hydrogen bonding with adjacent water molecules), while the peaks at 3436 and 3637 cm^−1^ are associated with weakly hydrogen‐bonded IW.^[^ ^34^ ^]^ Hence, the stronger IW peaks compared with FW peaks indicate a higher proportion of IW in the CFL hydrogel. Differential scanning calorimetry (DSC) further confirmed these findings, revealing that the CFL hydrogel exhibits a markedly smaller endothermic peak compared with deionized water (Figure 3n).^[^ ^35^ ^]^ This finding further confirms the presence of intermediate water within the CFL hydrogel, thereby lowering the energy input necessary for water evaporation. This reduction in energy requirement for water evaporation highlights the unique hydration structure of the hydrogel and its potential for efficient evaporation‐driven applications. The evaporator achieves high‐performance solar desalination through an integrated hydro‐thermal regulation mechanism. The hierarchically porous structure of CF aerogel facilitates rapid capillary‐driven water transport, while an outer foam layer significantly reduces heat dissipation. Simultaneously, the hydrophilic polymer network of CFL hydrogel modulates water molecular states by increasing the proportion of intermediate water, thereby lowering the evaporation enthalpy. The coordination between LS and Fe^3^⁺ enables broadband solar absorption, while the porous architecture of CFL hydrogel confines thermal energy at the evaporation interface. These synergistic effects collectively empower the all‐biomass evaporator to achieve highly efficient water evaporation.

**Figure 4:**
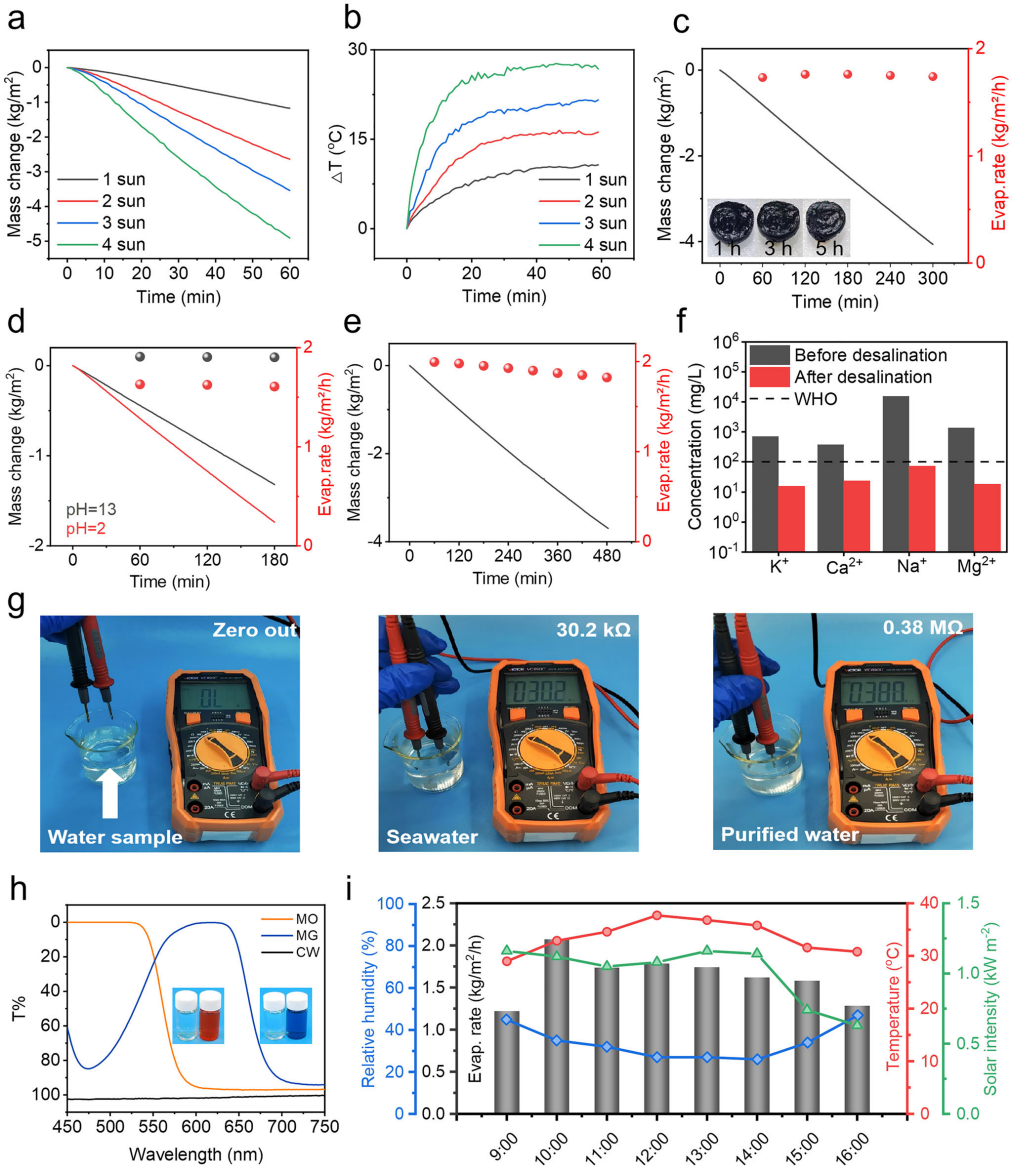
a) Water mass changes curves versus time with the steam generator under different radiation intensities (1, 2, 3, 4 sun) in fresh water. and b) the temperature evolution with the steam generator under identical conditions. c) water evaporation rates of the steam generator in 3.5 wt.% NaCl solution. d) evaporation rates of the steam generator in acid or alkali solutions (black corresponds to acidic conditions, red represents alkaline conditions). e) 8 h evaporation performance of the steam generator. f) the concentration of four main ions (K^+^, Ca^2+^, Na^+^, Mg^2+^) in seawater before and after desalination. g) The resistance of different water measured by a multimeter with constant electrode spacing. h) the transmittance of MO and MG aqueous solutions before and after desalination. i) the changes of ambient temperature, solar intensity, relative humidity and evaporation rate in outdoor experiment.

The evaporation performance was systematically evaluated under controlled laboratory conditions. Real‐time mass loss measurements, acquired using a high‐precision electronic balance, revealed a linear increase in evaporation rate with solar irradiance intensity (1‐4 sun). Concurrent infrared thermography measurements demonstrated stable thermal response characteristics across the tested radiation range. These results confirm the system's stable photothermal conversion efficiency and reliable operation under variable solar flux conditions (**Figure** **4**a,b). Importantly, the observed linear scaling relationship between evaporation rate and incident power density suggests highly efficient solar energy utilization, with no evidence of performance saturation within the tested intensity range.

A major limitation in the long‐term performance of solar evaporation systems is the progressive accumulation of salt on the photothermal interface. This phenomenon arises from a mismatch between the rate at which salt redissolves and the rate of water evaporation, ultimately leading to substantial crystallization on the photothermal surface. The formation of salt crystals not only increases the reflection of incident solar radiation—thereby reducing photothermal absorption efficiency—but also physically blocks the water transport channels essential for sustained evaporation. Together, these effects significantly compromise the operational stability and efficiency of the system over time. To investigate the system's resistance to salt accumulation, the CFL hydrogel was exposed to continuous solar irradiation (1 sun) in a 3.5 wt.% NaCl solution. Over a five‐hour illumination period, the water evaporation rate remained consistently stable at ≈1.75 kg m^−^^2^ h^−1^, with no visible salt crystallization detected on the photothermal surface (Figure 4c). The exceptional salt‐rejecting property of the evaporator is attributed to its rapid capillary‐driven water transport through the highly porous, interconnected network, which ensures a continuous and rapid supply of water from the bulk reservoir to the evaporation interface. This persistent convective flow effectively mitigates salt accumulation by dissolving and redispersing ions from the photothermal surface, thereby preventing localized supersaturation and crystallization. Furthermore, the interconnected pore structure facilitates efficient ion diffusion, continuously transporting ions away from the evaporation zone to suppress nucleation. To assess long‐term salt resistance and stability, we subjected the all‐biomass‐based solar evaporator under 1 sun illumination for a week in a 3.5 wt.% NaCl solution (Figure S11, Supporting Information). The evaporator also maintained a stable evaporation rate of ≈1.75 kg m^−^^2^ h^−1^ throughout the testing period, demonstrating exceptional resilience against salt accumulation and sustained performance. These findings underscore the exceptional salt‐tolerant properties of the system and demonstrate that the CF aerogel serves as an effective water transport scaffold, enabling sustained evaporation under saline conditions.

The system's tolerance to chemically extreme environments was further examined by evaluating evaporation performance in highly acidic (pH 2) and alkaline (pH 13) solutions. As illustrated in Figure 4d, under 1 sun irradiation, the steam generator maintained a steady evaporation rate of ≈1.91 kg m^−^^2^ h^−1^ over a three‐hour period in the pH 13 solution. Although a minor decline in rate was noted, the system continued to operate stably, demonstrating its resistance to alkaline‐induced degradation. These findings indicate that the steam generator retains structural integrity and functional performance under harsh pH environments. To assess the long‐term operational stability of the system, extended solar evaporation experiments were conducted. As shown in Figure 4e, the steam generator exhibited a linear water mass loss over an 8‐hour period under continuous 1 sun illumination, corresponding to an average evaporation rate of ≈1.91 kg m^−^^2^ h^−1^. The consistent rate throughout the test period highlights the system's excellent durability and operational stability under sustained solar exposure. To further validate the system's practical applicability, solar distillation was performed using natural seawater sourced from Dalian, Liaoning, China (longitude: 121.505 latitude: 39.029). The quality of the collected water was analyzed via inductively coupled plasma optical emission spectrometry (ICP‐OEC). As shown in Figure 4f, the concentrations of the four major ions‐Na⁺, Mg^2^⁺, K⁺, and Ca^2^⁺‐were reduced by approximately one to two orders of magnitude compared to those in the original seawater. These results indicate that the purified water produced by the steam generator approaches the quality standards set by the World Health Organization (WHO) for drinking water,^[^ ^36^ ^]^ underscoring the potential of the evaporator for real‐world desalination applications. Electrical measurements provided additional evidence. Further assessment of desalination performance was conducted by measuring the electrical resistance of seawater before and after purification, using a multimeter. The resistance increased markedly from 30.2 kΩ to 0.38 MΩ after treatment, reflecting a substantial reduction in ionic content and confirming the high efficacy of the steam generator in seawater purification (Figure 4g).

The wastewater treatment potential of the system was evaluated with two organic dyes: methyl orange (MO) and malachite green (MG). As shown in Figure 4h, transmittance measurements revealed that MO and MG solutions exhibited 0% light transmittance within the 450–550 nm and 500–700 nm spectral ranges, respectively, whereas the condensed water following solar evaporation demonstrated nearly 100% transmittance across the same regions.^[^ ^37^ ^]^ These results highlight the capacity of system to remove organic pollutants, thereby extending its functionality to sewage treatment. In outdoor testing (Figure 4i), the solar‐driven interfacial evaporator achieved evaporation rates between 1.0 and 2.0 kg m^−^^2^ h^−1^ when tested with simulated seawater under outdoor conditions, demonstrating consistently high performance in a natural environment. These results highlight the system's strong operational efficiency and suggest that the biomass‐based solar evaporator holds considerable promise for practical and scalable applications in solar‐driven desalination. In order to comprehensively evaluate the operational durability and environmental stability of our all‐biomass evaporator under realistic conditions, we conducted 100 consecutive evaporation–cooling cycles under one‐sun irradiation using natural seawater collected from the coastal area of Dalian (Figure S12, Supporting Information). The evaporator demonstrated remarkable stability, maintaining an average evaporation rate of ≈1.85 kg m^−^^2^ h^−1^ throughout the testing period. This outstanding cyclic performance can be attributed to the robust hydrophilic network formed through CNF–Fe^3^⁺ coordination and the exceptional photothermal conversion capability achieved by LS–Fe^3^⁺ coordination. The preservation of structural integrity over repeated hydration–dehydration cycles confirms the material's resistance to physical degradation in marine conditions. Furthermore, a seven‐day soil burial test was conducted to assess the environmental stability of the evaporator (Figure S13, Supporting Information). The material exhibited minimal mass loss (∼0.38%), indicating pronounced resistance to biodegradation. This robustness originates from its dense cross‐linked network, which effectively inhibits microbial infiltration. Coupled with its sustained evaporation performance in natural seawater, these results demonstrate the potential of the all‐biomass‐based evaporator for long‐term operation in real‐world environments.

**Figure 5:**
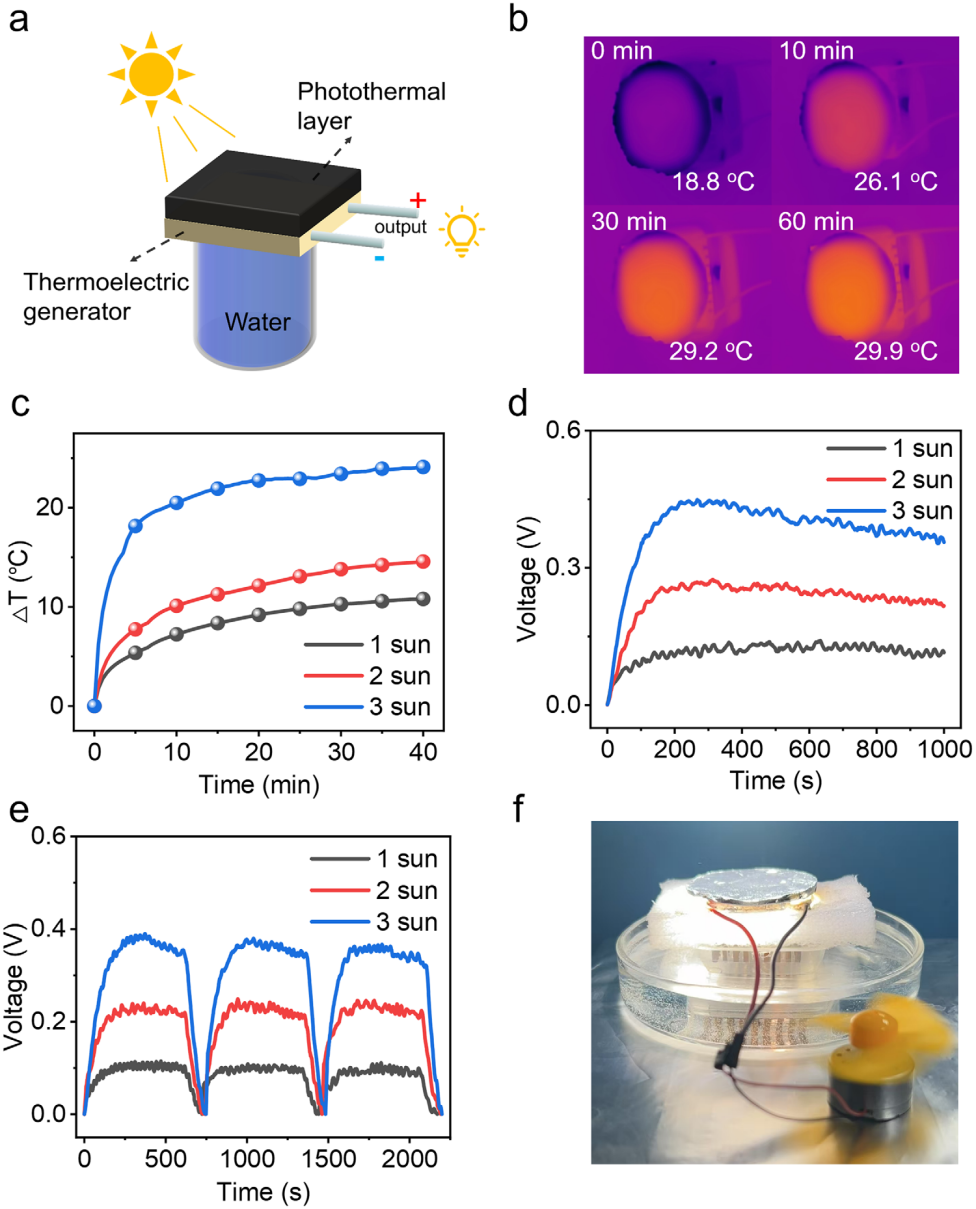
a) Digital image of PTE generator. b) Infrared image of surface temperature of the generator under 1 sun and 2 sun irradiations at 0, 10, 30, 60 min. c) Surface temperature evolution curves of the generator under 1, 2, 3 sun irradiation. d) Open‐circuit voltage of the PTE generator under 1, 2 and 3 sun. e) Open‐circuit voltage of the PTE generator under 1, 2 and 3 sun toward light cyclic responses. f) Digital photo of fan rotation.

The superior photothermal performance of CFL hydrogel induces a pronounced temperature gradient between its surface and the adjacent cold water during operation. Capitalizing on this thermal gradient, we developed a photothermal‐electric (PTE) generator capable of converting heat into electrical energy. This device harnesses the Seebeck effect to generate electricity by exploiting the temperature difference established across its two sides.^[^ ^38^ ^]^ As shown in **Figure** **5**a, when the PTE device floated on the water surface with support from a sponge, its top side was covered with a layer of CFL hydrogel to absorb solar energy, thereby creating the high‐temperature side, while the water served as the cold side. While the PTE device operated on the water surface, the open‐circuit voltage (Voc), short‐circuit output current (Isc), and current‐voltage curves were recorded using an electrical workstation. Due to the excellent photothermal properties of CFL hydrogel, under 1 sun irradiation level, the surface temperature of the PTE device increased from 18.8 to 29.9 °C after 60 min. Under 2, 3 sun irradiation, the surface temperature of the PTE device increased by 15.3 and 24.5 °C, respectively, after 60 min, further confirming the CFL hydrogel excellent photothermal response (Figure 5b,c).

The PTE device achieved maximum voltage outputs of 0.13, 0.26, and 0.44 V under 1, 2, and 3 sun irradiation, respectively. However, the output voltages exhibited a decreasing trend after reaching their maximum values and approached a stable state after 800 s (Figure 5d). The phenomenon was consistent with the previous reports from the other groups.^[^ ^39^ ^]^ Switching on the light by three on‐off cycles, as shown in Figure 5e, the PTE device produced output open‐circuit voltage. Figure 5f illustrates the capability of the PTE device to power a fan, demonstrating its stable and effective photo‐thermal‐electric energy conversion. These results underscore the potential of CFL hydrogel as a promising material for advancing photo‐thermo‐electric applications. An outdoor evaluation of the power generation system was conducted to assess its performance under realistic environmental conditions. Measurements were taken at 2‐h intervals from 7:00 to 17:00 under natural daylight illumination (Figure S14, Supporting Information). The voltage output of the thermoelectric module was recorded using a digital multimeter. The results indicate that the evaporator consistently generated electricity throughout the testing period, with the thermoelectric module producing voltages ranging from 50 to 72 mV. The experiment confirms the functional efficacy and practical viability of the device under real environmental conditions. A comparative performance analysis confirms the superior thermoelectric efficiency of our device against previously reported biomass‐derived interfacial evaporators (Figure S15 and Table S2, Supporting Information).

Finally, the manufacturing cost of the all‐biomass‐based solar‐driven interfacial evaporator was evaluated, confirming its economic feasibility with an estimated production cost of $59.87/m^2^ (Table S3, Supporting Information). Comparative analysis against previously reported evaporators demonstrates simultaneously superior cost‐effectiveness and enhanced evaporation performance, highlighting the scalability and practical potential of our design for sustainable desalination applications (Table S4, Supporting Information). Given the cost efficiency and operational simplicity of the proposed synthesis route, it can be readily integrated into existing industrial frameworks, thereby facilitating large‐scale production. The procedure is outlined as follows: A homogeneous CNF sol is prepared by dispersing CNF in deionized water under mechanical stirring. The sol is then injected under automated control into custom‐designed molds. After degassing, an FeCl_3_ solution is introduced via precision injection to initiate uniform cross‐linking, resulting in the formation of CF hydrogel. This hydrogel is subsequently converted into a porous aerogel (CF aerogel) through freeze‐drying device. Simultaneously, an aqueous mixture of LS and FeCl_3_ is prepared using mechanical agitation. The CF hydrogel is then immersed in this solution to yield a CFL hydrogel. Finally, the CF aerogel is encapsulated with structural foam using an automated foam‐filling machine and assembled into the final evaporator structure.

## 3 Conclusion

In summary, a 3D all biomass‐based solar‐driven interfacial evaporator was constructed. Fe^3^⁺‐LS coordination induced photothermal complexes exhibit exceptional solar absorption property (95%). The hydrophilic polymer network of hydrogel tunes the water into intermediate state, significantly reducing the evaporation enthalpy. Synergistically, the integrated porous CF aerogel and coordination‐enhanced CFL hydrogel establish hierarchical capillary networks that facilitate efficient water transport. Thus, the synergy of enhanced solar absorption, thermal management, water transport, and water activation yields a fast evaporation rate of 1.91 kg m^−2^ h^−1^. Furthermore, integration of the photothermal CFL hydrogel with thermoelectric generation modules achieve sufficient voltage output (110 mV) to drive fans. Collectively, this fully biomass‐based platform represents a promising approach for addressing interconnected challenges of water scarcity, energy poverty, and environmental sustainability.

## 4 Experimental Section

### Materials

CNF were provided by Guilin Qi Hong Technology Co. Ltd. Ferric chloride hexahydrate (FeCl_3_·6H_2_O) and Sodium Lignosulfonate (LS) were supplied by Macklin Co. Ltd.

### Preparation of CF Aerogel as Water Transport Channel

A homogeneous 2 wt.% CNF dispersion was obtained by magnetically stirring CNF in deionized water (1000 rpm, 1 h). In parallel, an aqueous FeCl_3_·6H_2_O solution was prepared by in deionized water under magnetic stirring (500 rpm, 30 min). The FeCl_3_ solution was introduced dropwise into the CNF dispersion at a 1:1 ratio, yielding an orange CF hydrogel after 6 h of reaction. The hydrogel was then pre‐frozen (−50 °C, 12 h) and lyophilized (72 h) to produce a porous CF aerogel.

### Preparation of CFL Hydrogel as Sunlight Absorber

A precursor solution was prepared by dissolving FeCl_3_·6H_2_O and LS in deionized water at a stoichiometric ratio of 1:5, with LS maintained at 5 wt.%. The mixture was subjected to continuous magnetic stirring (500 rpm, 30 min, 25 °C) until formation of a homogeneous black solution. The pre‐synthesized CF hydrogel matrix was then immersed in the LS/FeCl_3_ solution for 3 h under static conditions, resulting in the formation of the CFL hydrogel.

### Solar‐Driven Interfacial Evaporator Fabrication

Steam Generator Fabrication: The CF aerogel was placed in the center of a circular foam ring (with the size of the CF aerogel matching the inner size of the circular ring). The CFL hydrogel was treated by surface grinding or physical cutting methods to create a rough surface. Finally, the CFL hydrogel with the rough surface was placed above the foam to contact with CF aerogel, and the three components were integrated to form the evaporator.

### Characterization and Water Evaporation Measurements

The morphology and elemental mapping of the gels were analyzed using thermal field emission scanning electron microscope (JSM‐7800F,) with energy dispersive X‐ray (EDS) spectroscopy. The chemical composition of the samples was characterized by X‐ray photoelectron spectroscopy (Thermo Scientific K‐Alpha). Fourier transform infrared (FTIR) spectroscopy was employed to obtain the FTIR spectra. The water contact angle of the gels was measured using an optical contact angle meter. UV–vis spectroscopy (Shimadzu UV‐3600i Plus) equipped with an integrating sphere was used to measure the optical diffuse transmittance and reflection spectra of the gels. Absorbance was calculated using Kirchhoff's law as A = 1−R−T, where R and T represent reflection and transmission, respectively. The concentration of metal ions was measured with inductively coupled plasma‐optical emission spectrometry (ICP‐OES). Water evaporation performance experiments were conducted using Solar simulator (Solar‐500T). The solar flux was calibrated using a thermopile connected to a power meter. Real‐time mass changes of water were recorded using a high‐precision electronic balance (AE224C). The temperature distribution of the systems was monitored with an infrared camera (FOTRIC 226S) and infrared analysis software (AnalyzIR).

### Photothermal‐Electric Characterization

Different intensities of sunlight were simulated by adjusting the power of the sunlight simulator to irradiate the CFL hydrogel. The output voltage and current of the thermoelectric system were measured and recorded using software.

## Conflict of Interest

The authors declare no conflict of interest.

## Supporting information



Supporting Information

Supplemental Movie

## Data Availability

The data that support the findings of this study are available from the corresponding author upon reasonable request.
