# Supplementary material for: All Biomass‐Based Solar‐Driven Interfacial Evaporator for Efficient Seawater Desalination and Power Generation
Source: Adv Sci (Weinh). 2025 Sep 25;12(46):e13258. doi: 10.1002/advs.202513258 (PMC12697891; doi:10.1002/advs.202513258)
Supplement: Supplementary file 1 — Supporting Information [file ADVS-12-e13258-s001.docx]

**Supporting Information**

**Figure S1.** (a) Photographs of CFL gel with different shapes, (b) water mass change curves versus time with CFL gel of different shapes under 1 sun, (c) the transmittance of various lignin content, (d) mass change of water versus time with CFL gel of distinct ratio mass of Fe^3+^, (e) the temperature changes of CFL gel containing Fe^3+^ in different proportions under one sun irradiation, (f) the mass loss of CFL gel by soaking various ratio of Fe^3+^ and LS solution, (g) the temperature variation curve of CFL gel with different proportions of Fe and LS, (h) the CFL gel mass loss of soaking for different times, (i) changes in temperature of drowning different times.

The excellent photothermal conversion is beneficial to increase the evaporation rate. Therefore, conducting a series of explorations on the photothermal layer. The gel has high plasticity, so that it can be made into various shapes such as jelly, rose, shell, etc. (Figure S1a). This represents that Gel-based solar interface evaporators can be shaped differently to adapt to various environments. Then, different shapes of photothermal layers were used to test, in order to investigate the effect of different shapes on the evaporation rate. The curve graph indicates that linear water mass loss is achieved regardless of the shape of the photothermal layer, confirming the good stability of the evaporator (Figure S1b). However, it was observed that varying shapes had little impact on the rate, with a low evaporation rate, approximately 1.1 kg/m²/h, which did not reach the ideal rate. Previous reports on solar interface evaporators have suggested that increasing the surface roughness of photothermal layers helps to enhance light reflection and evaporation rates^[1]^. The results indicate that the square evaporative layer with a rough outer surface (Figure S1b). The evaporation rate showed a significant improvement, which can reach approximately 1.9 kg/m²/h in the subsequent experiment.

The preliminary verification demonstrated the evaporation capability of the light-absorbing layer, sparking interest in the optical properties of Sodium Lignosulfonate (LS). LS exhibited light-absorbing properties due to its structure, which includes benzene rings and conjugated systems. The optical properties of lignin were measured by an ultraviolet (UV)-visible (vis)-NIR spectrophotometer (200–1100 nm). As shown in Figure 5c, the results indicated that the transmittance decreased with the increase of lignin content. Previous studies have indicated that metal ions exhibit certain photothermal effects^[2]^. Thus, to investigate the influence of Fe^3+^ on the photothermal properties of a gel matrix. The impact of CNF soaking Fe^3+^ solutions with varying mass ratios and then gelation on evaporation was detected. CNF was gelated with Fe^3+^ solution of different mass ratios (0.5%, 1%, 2%, 5%). As shown in Figure S1d, e, the best photothermal effect was achieved by CNF gelation induced by 2% Fe^3+^, under the same conditions, the 2% Fe^3+^ sample increased approximately 18 ℃. The results displayed that the evaporative performance of the formation of CF gel in the first step is influenced by immersion in different Fe^3+^ solutions (Figure 6d). And figure 3e showed that the temperature rise trend stabilizes after approximately ten minutes.

In addition to exploring the photothermal performance during gelation, the mixture of lignin and Fe^3+^ is also crucial because of its increased blackness. Five Fe^3+^/LS solution with varied Fe^3+^:LS ratios (10:1 and from to 1:10), where the proportion of Fe^3+^ was fixed. The photothermal effect, heating rate, and evaporation rate all increased with the rise in lignin content (Figure S1 f, g). The darkening of the LS solution is attributed to the formation of coordination bonds between Fe^3+^ and the hydroxyl groups in LS. When CF gel was placed into a Fe^3+^/LS solution, LS gradually permeated into the gel (Figure 2). The influence of evaporation performance was measured for different soaking times. It was found that soaking for 3 hours yielded the best results, with an increase of about 19℃ under 1 sun in 60 minutes (Figure 5h, i). Taking into account the above, 2% Fe^3+^ was selected as the ideal proportion for gelation with CNF, the Fe:LS(1:5) solution served as photothermal layer of formation ratio and soaked for 3 hours was optimal.


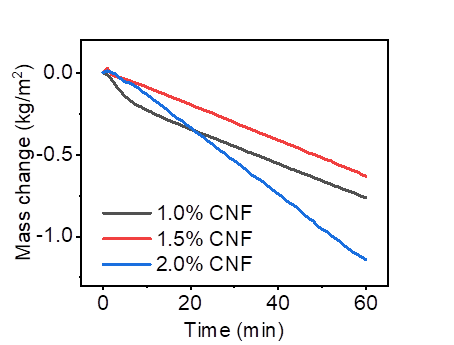


**Figure S2.** Mass change of water versus time with CFL gel of different ratio mass of CNF

Then, the gelation of CNF with varying concentrations was investigated, taking into account the presence of two main components responsible for gel formation (Figure S2). As the concentration of CNF increased, achieving thorough stirring became progressively challenging. Hence, only conditions with concentrations of 2% or below were taken into consideration. It was observed that Carboxyl cellulose nanofibers at a concentration of 2% stand out.


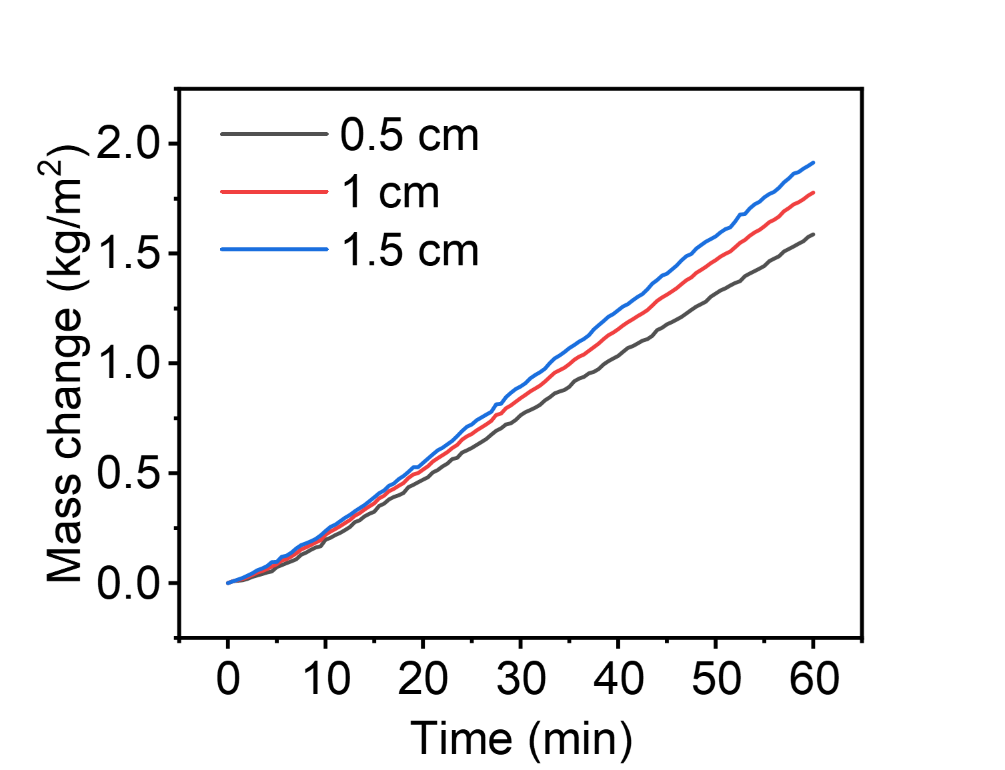


**Figure S3.** The CFL gel mass loss of different thickness.

To investigate the influence of photothermal layer thickness on evaporation efficiency, we performed controlled experiments under standard one-sun illumination using layers measuring 0.5, 1.0, and 1.5 cm in thickness. The results reveal a clear thickness-dependent enhancement in evaporation rate, with thicker layers consistently exhibiting superior performance.


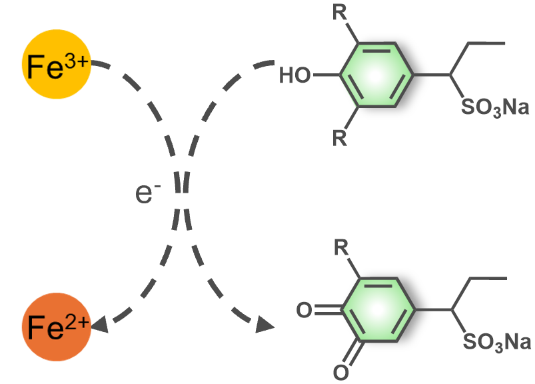


**Figure S4.** Schematic diagram of oxidation-reduction reaction


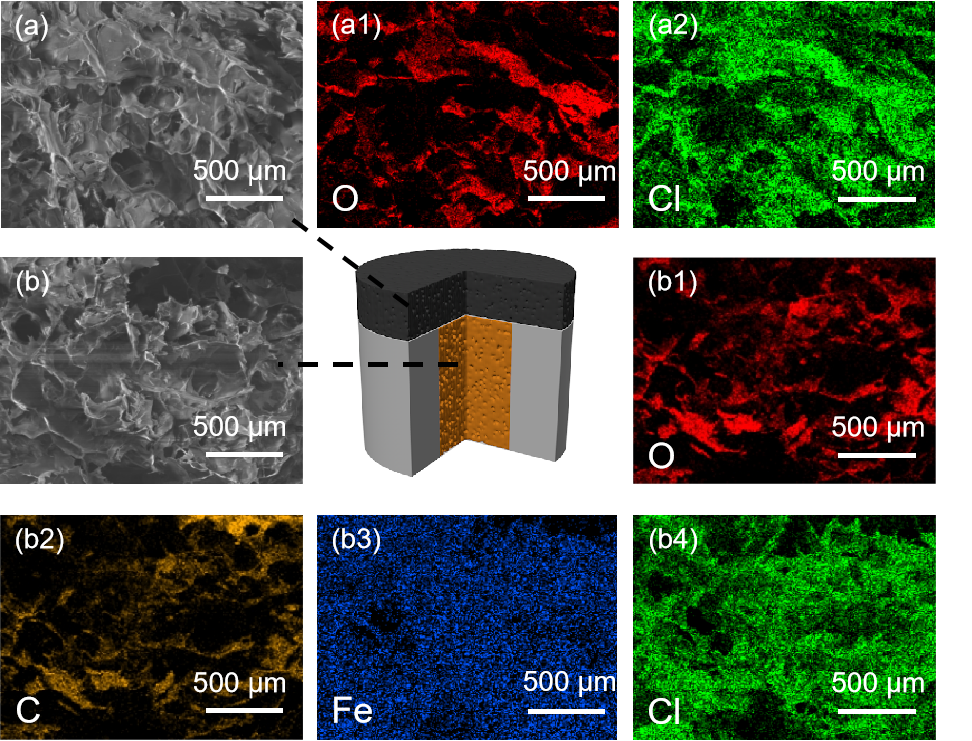


**Figure S5.** (a) SEM images of the interior view of CFL gel, (a1, 2) EDS mapping of the CFL gel, (b) SEM images of the internal structure of CF, (b1-4) EDS mapping of the CF gel.


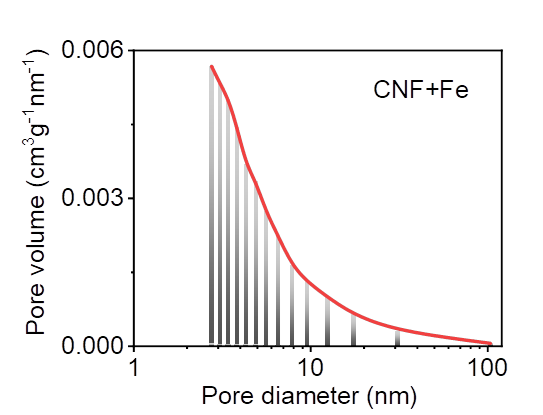


**Figure S6.** The pore size distribution curves of CF gel.


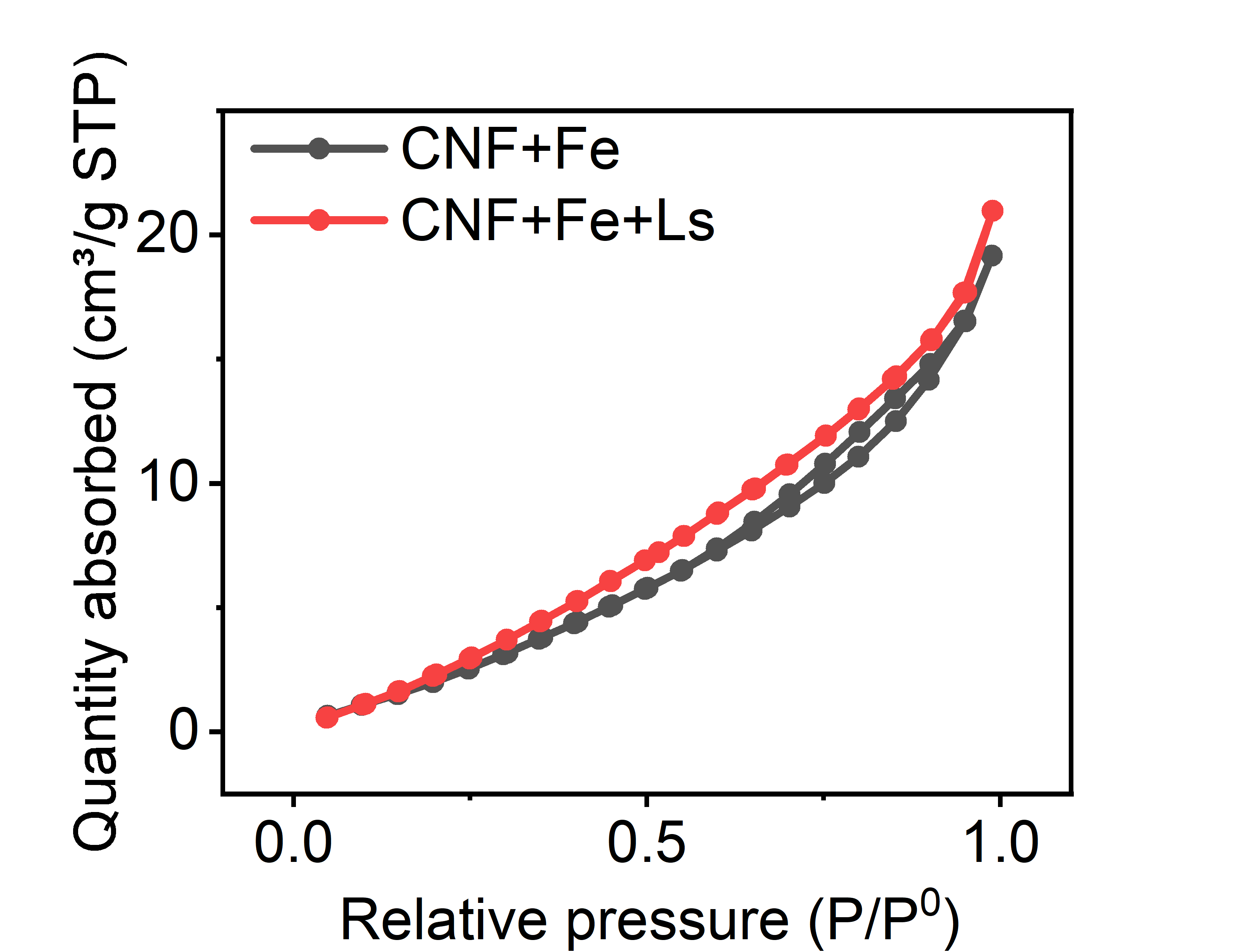


**Figure S7.** Nitrogen adsorption–desorption curve of CF and CFL gel.


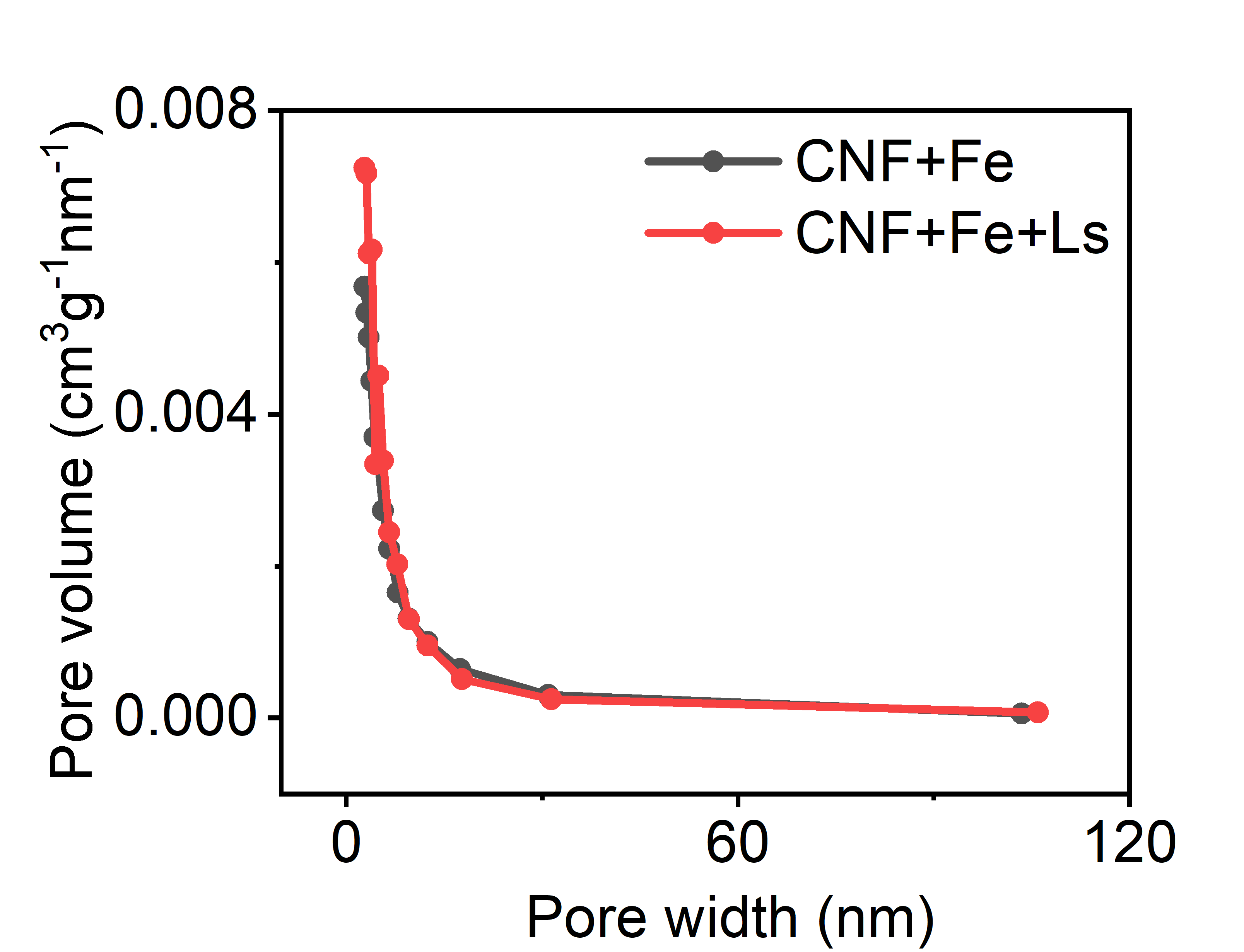


**Figure S8**. Pore-size distribution curve of CF and CFL gel.


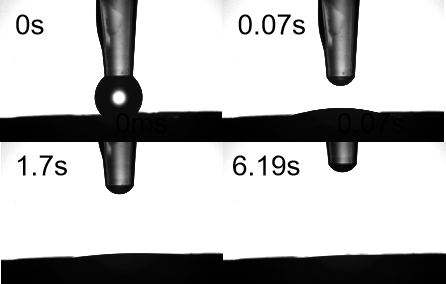


**Figure S9.** Optical image of the water dropped on the surface of CF hydrogel


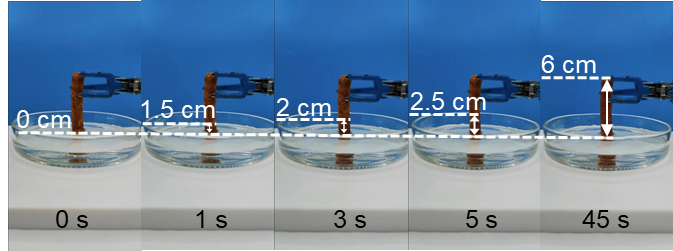


**Figure S10.** Digital picture of water transport.


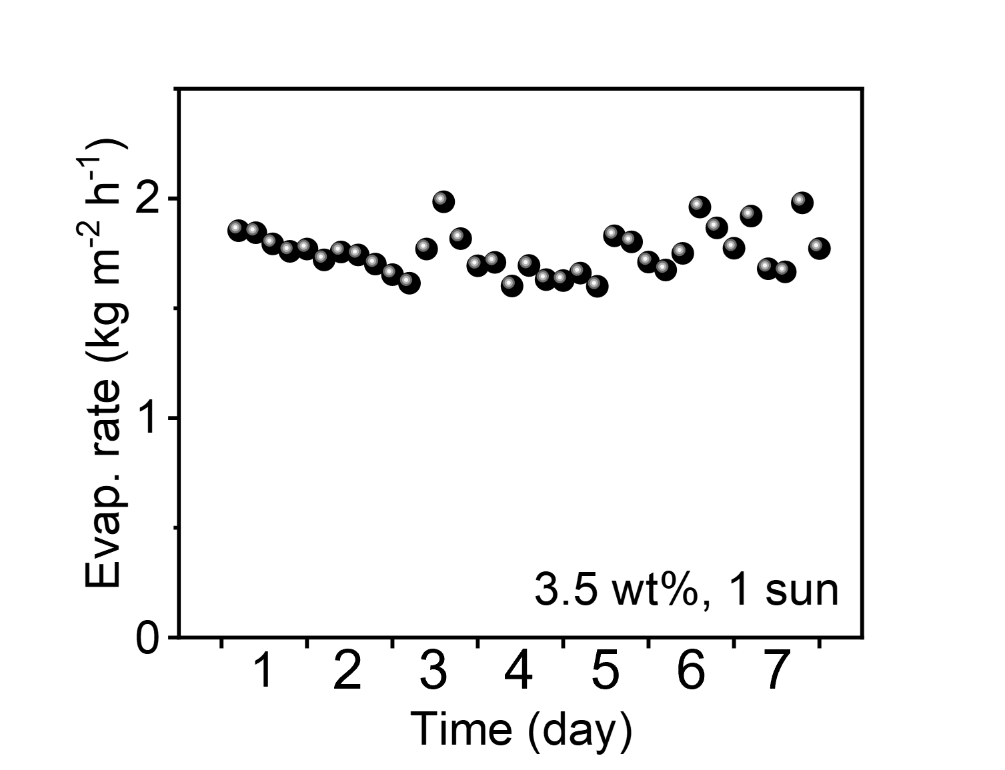


**Figure S11.** The evaporation rate of the evaporator in 3.5 wt% NaCl solution under 1 sun.


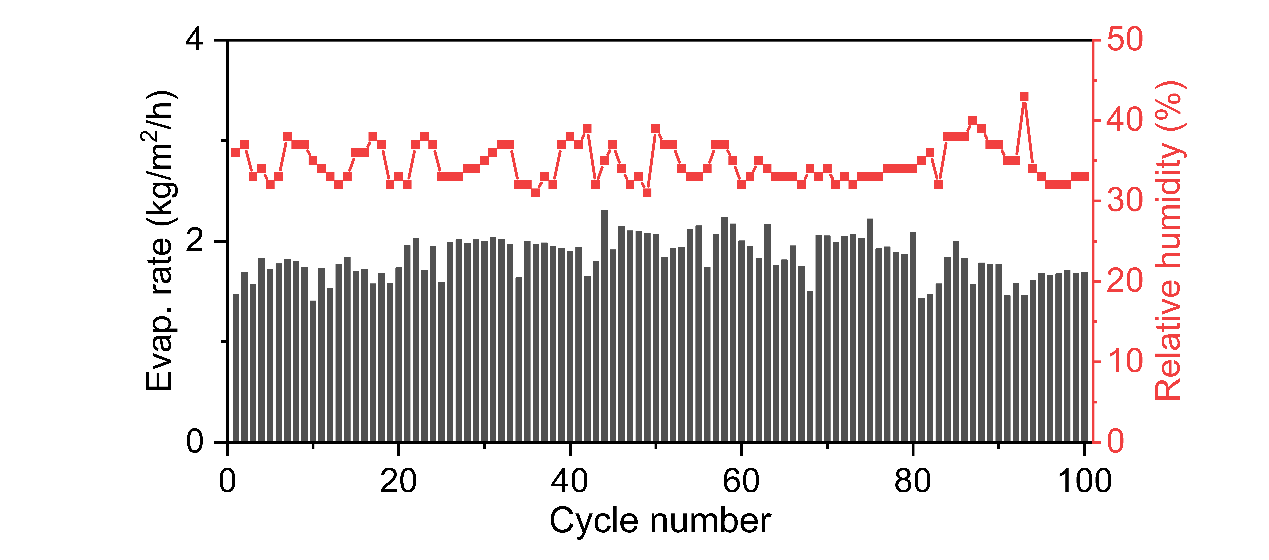


**Figure S12.** Evaporation rate of 100 evaporation cycles in real seawater under 1 sun.


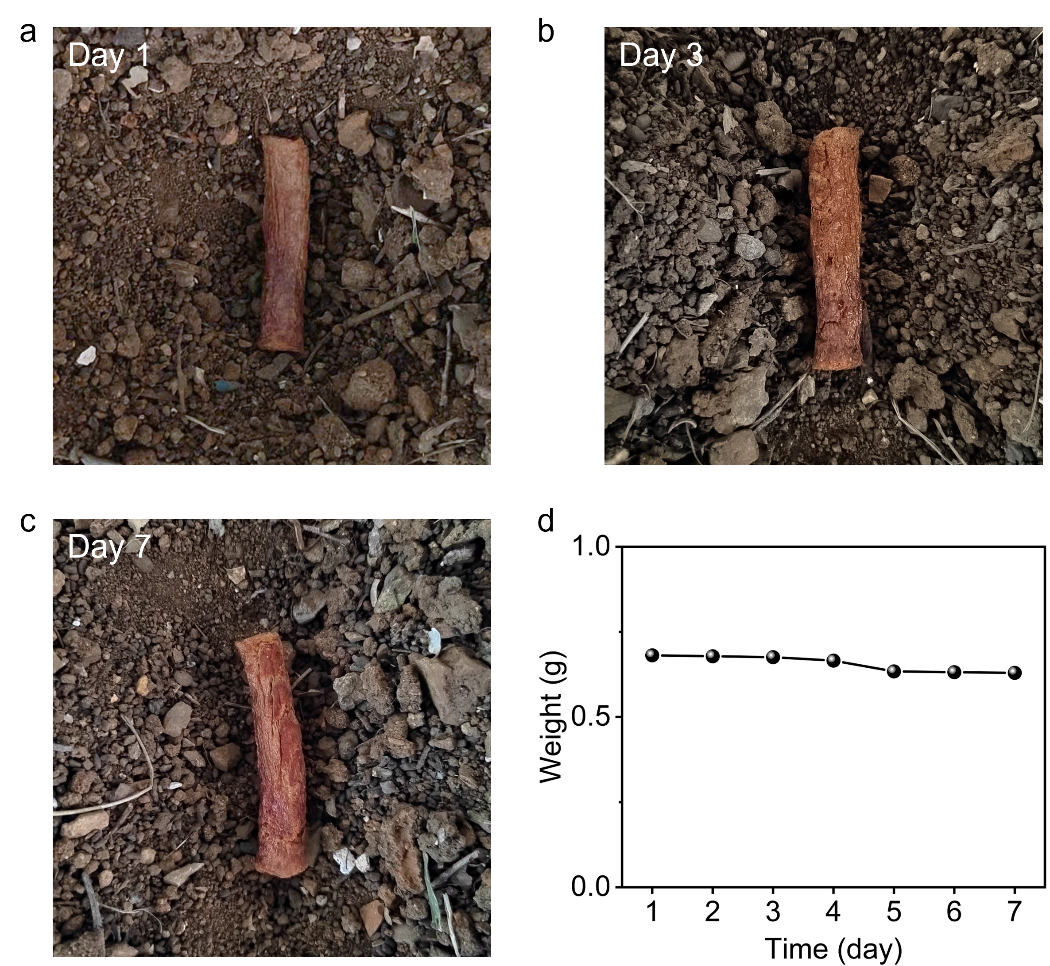


**Figure S13.** Photos of the material substrate of the evaporator in natural soil on (a) the first, (b) third, and (c) seventh day, (d) quality curve in degradation experiment.


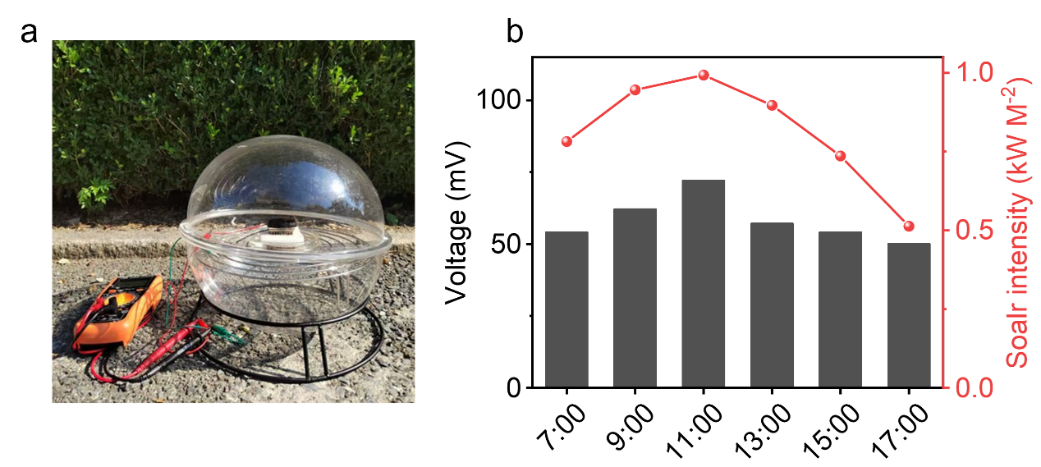


**Figure S14.** (a) Photos of outdoor power generation experimental equipment under natural sunlight, (b) record the output voltage (red, left axis) and solar intensity (red, right axis) of the evaporator surface under natural light every 2 hours, represented by a digital multimeter value.


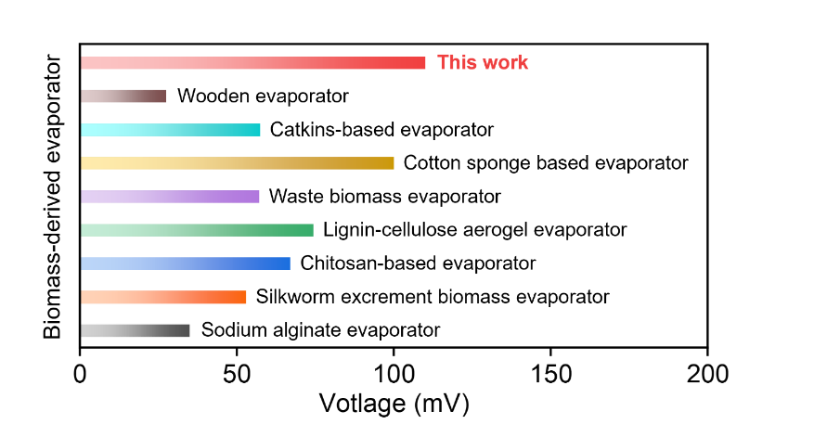


**Figure S15.** Comparison of the open-circuit voltage with previously reported biomass-derived evaporators under one sun irradiation.

**
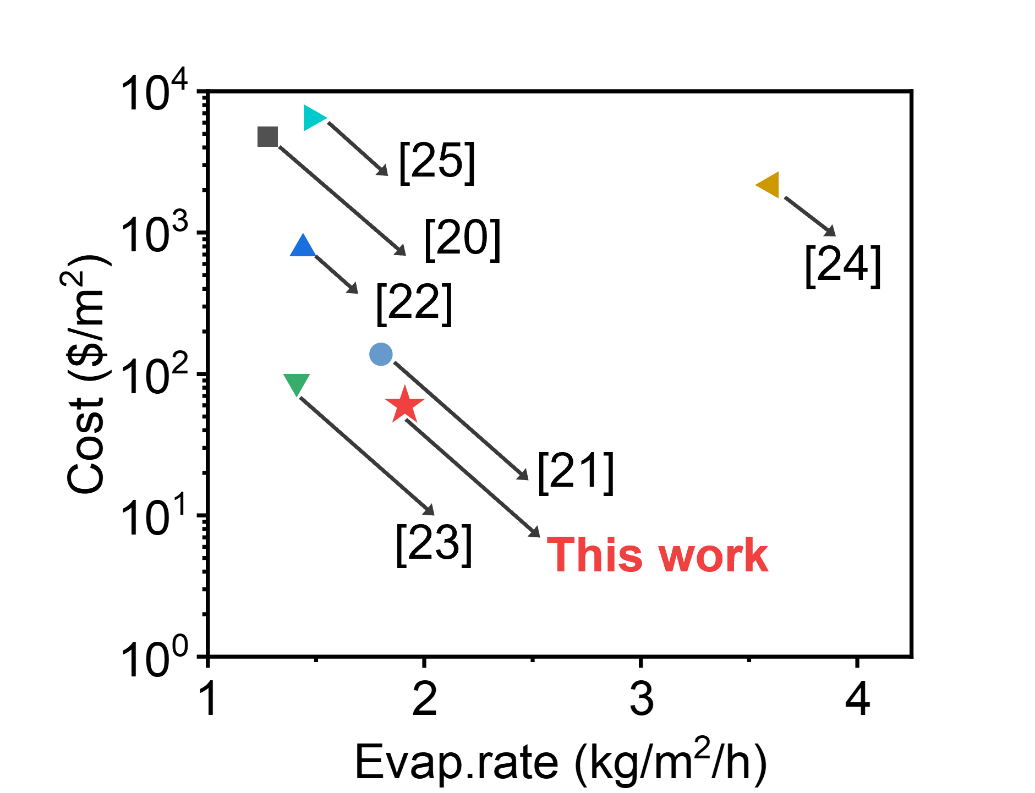
**

**Figure S16.** Comparison of materials costs and evaporation rates of solar evaporators with previously reported works.

| **Solar evaporator** | **Evaporation rate (kg/m^2^/h)** | **Ref.** |
| --- | --- | --- |
| Lignin carbon-chitosan evaporator | 1.717 | ^[3]^ |
| Carbonized vinegar residue evaporator | 1.45 | ^[4]^ |
| Wood-based anti-algae evaporator | 1.54 | ^[5]^ |
| Poplar-derived film | 1.77 | ^[6]^ |
| Carbonized peanut shell evaporator | 1.46 | ^[7]^ |
| Biomass-supported evaporator | 1.36 | ^[8]^ |
| Laser-engraved wood evaporator | 1.72 | ^[9]^ |
| Plantain cellulose evaporator | 1.86 | ^[10]^ |
| Lophius litulon-derived evaporator | 1.34 | ^[11]^ |
| All biomass-based solar-driven interfacial evaporator | 1.91 | This work |

**Table S1.** Comparison of solar steam generation performance of the all-biomass-based evaporator with previously reported biomass-derived evaporators under 1 sun irradiation in the past three years

| **Solar evaporator** | **Open-circuit voltage (mV)** | **Ref.** |
| --- | --- | --- |
| Sodium alginate evaporator | 34.93 | ^[12]^ |
| Silkworm excrement biomass evaporator | 53 | ^[13]^ |
| Chitosan-based evaporator | 67 | ^[14]^ |
| Lignin-cellulose aerogel evaporator | 74.43 | ^[15]^ |
| Waste biomass evaporator | 57.1 | ^[16]^ |
| Cotton sponge based evaporator | 100 | ^[17]^ |
| Catkins-based evaporator | 57.495 | ^[18]^ |
| Wooden evaporator | 27.5 | ^[19]^ |
| All biomass-based evaporator | 110 | This work |

**Table S2.** Comparison of the open-circuit voltage with previously reported biomass-derived evaporators under one sun irradiation.

| **Chemical** | **Purchase link** | **Price (CNY/kg)** | **Dosage (kg)** | **Cost (CNY/kg)** |
| --- | --- | --- | --- | --- |
| **CNF** | https://www.qh-tech.cn/CNF-C.html | 900 | 0.2608 | 234.72 |
| **Deionized water** | Locally marketed | 0.5 | 29.34 | 14.67 |
| **LS** | https://www.macklin.cn/products/S817764 | 305.5 | 0.489 | 149.39 |
| **FeCl_3_.6H_2_O** | https://www.macklin.cn/products/I809489 | 124.17 | 0.2282 | 28.34 |
| **All-in cost** | **¥430.12 /m^2^ （or $59.87 /m^2^）** | | | |

**Table S3.** Cost analysis of this work.

| **Solar evaporator** | **Material cost** | **Evaporation rate (kg m^−2^ h^−1^)** | **Ref.** |
| --- | --- | --- | --- |
| PDA@Mxene based evaporator | $4,778.26 /m^2^ | 1.276 | ^[20]^ |
| Porous carbon/paper pulp fiber membrane-based evaporator | $138.10 /m^2^ | 1.80 | ^[21]^ |
| Polyacrylonitrile/CNT nonwoven fabrics-based evaporator | $773.36 /m^2^ | 1.44 | ^[22]^ |
| PPy-coated air laid paper- based evaporator | $88.2 /m^2^ | 1.41 | ^[23]^ |
| PVA/CS/PPy - hydratable solar evaporator | $2174.27 /m^2^ | 3.6 | ^[24]^ |
| NiPS_3_/PVA-3D photothermal evaporator | $6480.06 /m^2^ | 1.48 | ^[25]^ |
| All biomass-based solar-driven interfacial evaporator | $59.87 /m**^2^** | 1.91 | This work |

**Table S4**. Comparison of materials costs of solar evaporators with previously reported works.

[1] B. Ma, F. Xiong, H. Wang, M. Wen, J. Yang, Y. Qing, F. Chu, Y. Wu, *Journal of Cleaner Production* **2024**, 435, 140506.

[2] A. O. Govorov, H. H. Richardson, *Nano Today* **2007**, 2, 30.

[3] S. Chen, B. Yang, D. Yang, X. Qiu, D. Zheng, *ACS Nano* **2025**, 19, 19681.

[4] H. Cao, H. Wang, X. He, Y. Yang, T. Ren, L. Liu, Y. Zheng, J. Fu, H. Zhou, *Journal of Materials Chemistry A* **2025**, 13, 23733.

[5] K. Zhu, C. Ma, L. Song, L. Jin, J. Wang, J. Wei, Y. Wu, X. Zheng, S. Wu, Y. Pang, Z. Shen, H. Chen, *Journal of Colloid and Interface Science* **2025**, 699, 138298.

[6] J. Sun, Y. Xin, Z. Li, B. Sun, X. Fan, *Chemical Engineering Journal* **2024**, 484, 149669.

[7] H. Cao, D. Li, J. Fu, Y. Yang, Y. Zheng, Y. Que, X. He, H. Wang, J. Liu, H. Zhou, *Desalination* **2024**, 591, 118052.

[8] S. Qi, L. Yuan, S. Ao, L. Wang, T. Jia, C. Dou, *Journal of Materials Chemistry A* **2024**, 12, 6663.

[9] Y. Pang, X. Chu, L. Song, L. Jin, C. Ma, Y. Wu, L. Li, Y. Peng, X. Zheng, F. Wang, S. Wu, Z. Shen, H. Chen, *Chemical Engineering Journal* **2024**, 479, 147891.

[10] J. Wu, X. Yang, X. Jia, J. Yang, X. Miao, D. Shao, H. Song, Y. Li, *Chemical Engineering Journal* **2023**, 471, 144684.

[11] J. Wang, Z. Zhao, C. Yang, M. Sun, J. Chen, Y. Zhou, H. Xu, *Desalination* **2023**, 556, 116577.

[12] Y. Long, X. Li, Y. Li, L. Wang, H. Zhu, G. Shi, *Chemical Engineering Journal* **2024**, 494, 152615.

[13] S. Ao, Z. Chen, H. Chen, L. Zhang, Y. Wang, Y. Zhang, T. Jia, G. Xi, T. Peng, *Journal of Materials Chemistry A* **2025**, 13, 15149.

[14] W. Zhou, F. Liu, Z. Li, X. Jing, T. Abdiryim, F. Xu, J. You, Y. Tan, X. Liu, *Chemical Engineering Journal* **2025**, 519, 165500.

[15] Z. Wei, C. Cai, Y. Huang, Y. Wang, Y. Fu, *Nano Energy* **2021**, 86, 106138.

[16] B. Fu, Y. Liu, Z. Jin, W. Guo, H. Shi, J. Luo, Y. Wang, F. Wang, T. Jia, X. Zhang, *Advanced Sustainable Systems* **2025**, n/a, e00659.

[17] T. Chen, Y. Tang, N. Song, Z. Lin, L. Xu, X. Peng, X. Chen, M. He, *Advanced Sustainable Systems* **2025**, 9, 2400618.

[18] J. Li, M. Liu, Y. Cui, W. Luo, Y. Jing, G. Xing, H. Sun, Z. Zhu, W. Liang, A. Li, *Surfaces and Interfaces* **2023**, 41, 103170.

[19] W. Lu, D. Jiang, Z. Wang, X. Zhang, Q. Ding, Z. Zhang, X. Liu, L. Bai, Z. Li, Y. Liu, *Chemical Engineering Journal* **2024**, 496, 154361.

[20] X. Zhao, X.-J. Zha, L.-S. Tang, J.-H. Pu, K. Ke, R.-Y. Bao, Z.-y. Liu, M.-B. Yang, W. Yang, *Nano Research* **2020**, 13, 255.

[21] L. Hao, N. Liu, R. Niu, J. Gong, T. Tang, *Science China Materials* **2022**, 65, 201.

[22] B. Zhu, H. Kou, Z. Liu, Z. Wang, D. K. Macharia, M. Zhu, B. Wu, X. Liu, Z. Chen, *ACS Applied Materials & Interfaces* **2019**, 11, 35005.

[23] C. Wang, Y. Wang, X. Song, M. Huang, H. Jiang, *Advanced Sustainable Systems* **2019**, 3, 1800108.

[24] X. Zhou, F. Zhao, Y. Guo, B. Rosenberger, G. Yu, *Science Advances*, 5, eaaw5484.

[25] H. Wang, Y. Bo, M. Klingenhof, J. Peng, D. Wang, B. Wu, J. Pezoldt, P. Cheng, A. Knauer, W. Hua, H. Wang, P. A. van Aken, Z. Sofer, P. Strasser, D. M. Guldi, P. Schaaf, *Advanced Functional Materials* **2024**, 34, 2310942.
